# Doenças do Aparelho Circulatório em Indivíduos com COVID-19: Descrição do Perfil Clínico e Epidemiológico de 197 Óbitos

**DOI:** 10.36660/abc.20200453

**Published:** 2020-08-19

**Authors:** Carlos Dornels Freire de Souza, Thiago Cavalcanti Leal, Lucas Gomes Santos

**Affiliations:** 1 Universidade Federal de Alagoas Campus Arapiraca Arapiraca AL Brasil Universidade Federal de Alagoas - Campus Arapiraca, Arapiraca, AL - Brasil

**Keywords:** Coronavírus-19/complicações, Febre, Síndrome Respiratória Aguda Grave, Dispneia, Transtornos Respiratórios, Fatores de Risco, Hipertensão, Diabetes Mellitus

**Caro Editor,**

Em dezembro de 2019, a China reportou à Organização Mundial da Saúde (OMS) um surto de pneumonia na cidade de Wuhan, capital da província de Hubei. Poucos dias depois, o agente causador foi identificado, o *severe acute respiratory syndrome coronavirus* 2 (SARS-CoV-2).^1^

A doença, batizada de coronavirus disease 2019 (COVID-19), rapidamente se espalhou pelos países e, em 11 de março de 2020, a OMS declarou pandemia global.^1^ No Brasil, o primeiro caso foi confirmado em 26 de fevereiro e a primeira morte em 17 de março, em São Paulo. Em 09 de abril, o Brasil somava 155.000 casos confirmados e 10.000 mortes pela doença.^2^

Os recentes estudos já apontam a dupla relação entre o aparelho circulatório e a COVID-19:^3 - 5^ i. o vírus pode causar alterações cardiovasculares, como arritmias, lesão cardíaca aguda, miocardite, dentre outras; ii. a presença de doenças do aparelho circulatório eleva o risco de agravamento e mortalidade pela COVID-19. Essa relação tem sido motivo de preocupação por parte de clínicos e cientistas. Partindo deste pressuposto, este estudo objetivou descrever o perfil clínico e epidemiológico de óbitos por COVID-19 que tinham doenças do aparelho circulatório previamente.

Trata-se de um estudo observacional transversal envolvendo 197 óbitos por COVID-19 ocorridos em Pernambuco que tinham pelo menos uma doença do aparelho circulatório previamente. Foram analisadas as seguintes variáveis: sexo, faixa etária, sinais/sintomas, comorbidades e fatores risco e tempo entre os primeiros sintomas e o óbito. Os dados foram obtidos da página eletrônica de monitoramento da COVID-19 do estado (https://dados.seplag.pe.gov.br/apps/corona.html) em 07 de maio de 2020. Após a coleta, o banco de dados passou por ajustes das variáveis para a subsequente análise. Neste estudo, foi utilizada apenas a estatística descritiva (frequência absoluta, frequência relativa, média e desvio padrão) com o auxílio do software SPSS versão 24.0 (IBM Corporation). Por utilizar dados de domínio público, este estudo dispensou a aprovação pelo Comitê de Ética em Pesquisa.

Em 07 de maio de 2020, o estado de Pernambuco já havia registrado 9.325 casos e 749 óbitos em decorrência da COVID-19. Desses óbitos, 293 (39,11%) possuíam o campo “ *comorbidades* ” preenchido (12 informavam que os pacientes não possuíam comorbidades e 281 citando as comorbidades). Dos 281 com comorbidades relatadas, 197 apresentavam pelo menos uma doença do aparelho circulatório, o que correspondeu a 70,10% dos indivíduos com comorbidades relatadas e a 26,30% de todos os óbitos.

Observou-se predomínio do sexo feminino (53,3%; n = 105) e de indivíduos com 50 anos ou mais (92,3%; n = 182). Os seguintes quatro sinais/sintomas apresentaram frequência superior a 50%: dispneia (80,7%; n = 159), tosse (72,1%; n = 142), febre (67,0%; n = 132) e saturação de oxigênio < 95% (58,9%; n = 116) ( Tabela 1 ).

**Tabela 1:** Caracterização clínica e epidemiológica dos óbitos por COVID-19 em Pernambuco com doenças do aparelho circulatório. Pernambuco, Brasil, 2020 (n=197)

Variável	n	%
**Sexo**		

Feminino	105	53,3
Masculino	92	46,7

**Idade (anos)**		

20-29	2	1,0
30-39	3	1,5
40-49	10	5,2
50-59	26	13,2
60-69	53	26,9
70-79	58	29,4
80+	45	22,8

**Sinais/sintomas**		

Febre	132	67,0
Dispneia	159	80,7
Tosse	142	72,1
Saturação de oxigênio < 95%	116	58,9
Dor de garganta	12	6,1
Astenia	9	4,6
Diarreia	9	4,6
Náuseas/vômito	7	3,6
Cefaleia	3	1,5
Mialgia	3	1,5
Perda de Peso	2	1,0
Dor abdominal	1	0,5
Coriza	1	0,5
Prisão de ventre	1	0,5

**Nº comorbidades**		

Somente uma	42	21,3
Duas	81	41,1
Três ou mais	74	37,6

**Sistema cardiovascular e hematológico**		

Hipertensão arterial sistêmica	163	82,7
Cardiopatia não especificada	51	25,9
Insuficiência venosa crônica	5	2,5
Doença arterial coronariana	4	2,0
Trombose	3	1,5
Arritmia	2	1,0
Doença de Chagas	1	0,5
Anemia	1	0,5

**Sistema endócrino/metabólico**		

Diabetes mellitus	106	53,8
Obesidade	22	11,2
Dislipidemia	2	1,0
Hipotireoidismo	1	0,5

**Sistema respiratório**		

Tabagismo	15	7,6
Doença pulmonar obstrutiva crônica	9	4,6
Pneumonia	2	1,0
Tuberculose	1	0,5
Asma	1	0,5

**Sistema neurológico**		

Acidente vascular cerebral (evento prévio)	16	8,1
Doença mental não especificada	5	2,5
Doença neurológica não especificada	4	2,0
Alzheimer	4	2,0
Miastenia	1	0,5

**Outras condições/fatores de risco**		

Doença renal crônica	21	10,7
Câncer	8	4,1
Etilismo	5	2,5
Doença dermatológica não especificada	3	1,5
Doença hepática	2	1,0
Amputação de membro	3	1,5
Outras condições com apenas um registro (HIV, pancreatite, transplante prévio, osteoporose, restrito ao leito).	1	0,5

**Tempo entre início dos sintomas e o óbito (média e desvio padrão, em dias)**	**9,7±7,8**	

Quanto à presença de comorbidades, 78,7% (n = 155) apresentavam duas ou mais comorbidades, sendo pelo menos uma relacionada ao aparelho circulatório. Dentre elas, a hipertensão arterial sistêmica foi observada em 82,7% (n = 163) dos indivíduos e a cardiopatia não especificada em 25,9% (n = 51) dos indivíduos ( Tabela 1 ).

Além do comprometimento do aparelho circulatório, as doenças e fatores de risco mais comuns na população estudada foram diabetes mellitus (53,8%; n = 106), obesidade (11,2%; n = 22), doença renal crônica (10,7%; n = 21), acidente vascular cerebral prévio (8,1%; n = 16), tabagismo (7,6%; n = 15), doença pulmonar obstrutiva crônica (4,6%; n = 9) e câncer (4,1%; n = 8). O tempo médio (em dias) entre o início dos sintomas e o óbito foi de 9,7 ± 7,8. Salienta-se que dos 197 pacientes incluídos neste estudo, em 10 deles não foi possível calcular o tempo entre o início dos primeiros sintomas e o óbito ( Tabela 1 ).

O perfil evidenciado neste estudo está em consonância com o observado em outras partes do mundo.^3 - 5^ No entanto, três aspectos chamam a atenção: i. a elevada proporção de indivíduos com múltiplas comorbidades (78,7% dos pacientes possuíam duas ou mais doenças/fatores de risco de base), ii. a ampla variedade de doenças/fatores de risco observado e iii. o estado clínico em que indivíduos chegaram ao atendimento hospitalar (função respiratória comprometida).

O somatório de comorbidades/fatores de risco em uma única pessoa pode elevar o risco de mortalidade pela COVID-19, embora ainda não existam estimativas precisas destes riscos. Em estudo conduzido em um hospital na cidade de Wuhan, China, envolvendo 416 pacientes internados com COVID-19, 44 (10,6%) e 22 (5,3%) tinham doença cardíaca coronariana e doença cerebrovascular, respectivamente. Outras comorbidades também foram observadas, como insuficiência cardíaca crônica (4,1%; n = 17), insuficiência renal crônica (3,4%; n = 14), doença pulmonar obstrutiva crônica (2,9%; n = 12) e câncer (2,2%; n = 9), assim como foi observado em nossa investigação. A mortalidade foi maior nos indivíduos com injúria cardíaca (51,2% no grupo com injúria vs. 4,5% no grupo sem injúria) e doenças preexistentes foram fatores associados à maior mortalidade.^4^

A associação entre várias comorbidade/fatores de risco pode explicar o quadro de comprometimento respiratório no momento da admissão, com dispneia e saturação de oxigênio < 95%, o que indica grave comprometimento pulmonar desses pacientes. A íntima relação funcional entre os sistemas cardiovascular (duplamente comprometido pela doença de base e pela infecção por SARS-CoV-2) e pulmonar (injúria pulmonar acentuada) deve ser valorizada no processo de cuidado dos pacientes com COVID-19 que apresentam doenças do aparelho circulatório.^3 - 5^

Por fim, destacamos a necessidade de adotar e/ou fortalecer mecanismos que reduzam a contaminação de indivíduos com doenças do aparelho circulatório pela COVID-19. Para aqueles já contaminados, o diagnóstico precoce e o monitoramento do quadro clínico devem ser rigorosamente observados, de modo a evitar o agravamento e a morte desses indivíduos.
