# Supplementary material for: Doenças do Aparelho Circulatório em Indivíduos com COVID-19: Descrição do Perfil Clínico e Epidemiológico de 197 Óbitos
Source: Arq Bras Cardiol. 2020 Aug 19;115(2):281–3. [Article in Portuguese] doi: 10.36660/abc.20200453 (PMC8384269; doi:10.36660/abc.20200453)
Supplement: Translation

# Translation: Circulatory System Diseases in Patients with COVID-19: Description of Clinical and Epidemiological Profile of 197 Deaths

**Authors:** Carlos Dornels Freire de Souza, Thiago Cavalcanti Leal, Lucas Gomes Santos

**Affiliations:** 1 Brazil Universidade Federal de Alagoas - Campus Arapiraca, Arapiraca, AL - Brazil

**Keywords:** Coronavirus-19/complications, Fever, Severe Acute Respiratory Syndrome, Dyspnea, Respiration Disorders, Risk Factors, Hypertension, Diabetes Mellitus

**Dear Editor,**

On December 31, 2019, China notified the World Health Organization (WHO) of an outbreak of pneumonia in the city of Wuhan, capital of the Hubei province. A few days later, the causative agent, severe acute respiratory syndrome coronavirus 2 (SARS-CoV-2) was identified.^1^

The disease, named coronavirus disease 2019 (COVID-19), rapidly spread throughout countries worldwide, and, on March 11, 2020, the WHO declared a global pandemic.^1^ In Brazil, the first case was confirmed on February 26, and the first death occurred on March 17, in São Paulo. On April 9, Brazil had a total of 155,000 confirmed cases and 10,000 deaths from the disease.^2^

Recent studies have already indicated the following double relationship between the circulatory system and COVID-19:^3 - 5^ i. The virus may cause cardiovascular changes, such as arrhythmias, acute cardiac injury, myocarditis, and others, and ii. The presence of circulatory system diseases increases the risk of worsening and mortality due to the disease. This relationship has been cause for concern on the part of clinical physicians and scientists. Based on this assumption, this study aimed to describe the clinical and epidemiological profile of deaths due to COVID-19 in patients who had prior circulatory system diseases.

This is a cross-sectional observational study involving 197 patients who died from COVID-19 in Pernambuco, all of whom had at least one prior circulatory system disease. The following were analyzed: sex, age range, signs/symptoms, comorbidities and risk factors, and time from first symptoms to death. Data were obtained from the state’s COVID-19 monitoring webpage (https://dados.seplag.pe.gov.br/apps/corona.html) on May 7, 2020. After collection, the database underwent adjustment of variables for subsequent analysis. In this study, only descriptive statistics (absolute frequency, relative frequency, mean, and standard deviation) were used, with the assistance of SPSS software, version 24.0 (IBM Corporation). Given that this study used public domain data, Research Ethics Committee approval was waived.

On May 7, 2020, the state of Pernambuco had registered 9,325 cases and 749 deaths due to COVID-19. Of these deaths, 293 (39.11%) had the field “comorbidities” filled out (12 stated that the patients had no comorbidities, and 281 cited the comorbidities). Of the 281 comorbidities reported, 197 presented at least one circulatory system disease, accounting for 70.10% of individuals with reported comorbidities and 26.30% of all deaths.

Predominance was observed in women (53.3%; n = 105) and patients 50 years or older (92.3%; n = 182). The following four signs/symptoms had frequency above 50%: dyspnea (80.7%; n = 159), cough (72.1%; n = 142), fever (67.0%; n = 132), and oxygen saturation < 95% (58.9%; n = 116) ( Table 1 ).

**Table 1:** Clinical and epidemiological characteristics of patients with circulatory system diseases who died from COVID-19 in Pernambuco. Pernambuco, Brazil, 2020 (n = 197)

Variable	n	%
**Sex**		

Female	105	53.3
Male	92	46.7

**Age (years)**		

20-29	2	1.0
30-39	3	1.5
40-49	10	5.2
50-59	26	13.2
60-69	53	26.9
70-79	58	29.4
80+	45	22.8

**Signs/symptoms**		

Fever	132	67.0
Dyspnea	159	80.7
Cough	142	72.1
Oxygen saturation < 95%	116	58.9
Sore throat	12	6.1
Asthenia	9	4.6
Diarrhea	9	4.6
Nausea/vomiting	7	3.6
Headache	3	1.5
Myalgia	3	1.5
Weight loss	2	1.0
Abdominal pain	1	0.5
Runny nose	1	0.5
Constipation	1	0.5

**Number of comorbidities**		

Only one	42	21.3
Two	81	41.1
Three or more	74	37.6

**Cardiovascular and hematological system**		

Systemic arterial hypertension	163	82.7
Unspecified heart disease	51	25.9
Chronic venous insufficiency	5	2.5
Coronary artery disease	4	2.0
Thrombosis	3	1.5
Arrhythmia	2	1.0
Chagas disease	1	0.5
Anemia	1	0.5

**Endocrine/metabolic system**		

Diabetes mellitus	106	53.8
Obesity	22	11.2
Dyslipidemia	2	1.0
Hypothyroidism	1	0.5

**Respiratory system**		

Tobacco use	15	7.6
Chronic obstructive pulmonary disease	9	4.6
Pneumonia	2	1.0
Tuberculosis	1	0.5
Asthma	1	0.5

**Neurological disease**		

Stroke (prior event)	16	8.1
Unspecified mental disease	5	2.5
Unspecified neurological disease	4	2.0
Alzheimer’s	4	2.0
Myasthenia	1	0.5

**Other conditions/risk factors**		

Chronic renal disease	21	10.7
Cancer	8	4.1
Alcoholism	5	2.5
Unspecified dermatological disease	3	1.5
Liver disease	2	1.0
Amputated limb	3	1.5
Other conditions with only one reported case (HIV, pancreatitis, prior transplant, osteoporosis, bedridden).	1	0.5

**Time from onset of symptoms to death (average and standard deviation, in days)**	**9.7 ± 7.8**	

With respect to the presence of comorbidities, 78.7% (n = 155) had two or more, at least one of which was related to the circulatory system. Systemic arterial hypertension was observed in 82.7% (n = 163) of individuals, and non-specified heart disease was observed in 25.9% (n = 51) ( Table 1 ).

In addition to involvement of the circulatory system, the most common diseases and risk factors in the study population were diabetes mellitus (53.8%; n = 106), obesity (11.2%; n = 22), chronic renal disease (10.7%; n = 21), prior stroke (8.1%; n = 16), tobacco use (7.6%; n = 15), chronic obstructive pulmonary disease (4.6%; n = 9), and cancer (4.1%; n = 8). Average time from onset of symptoms to death was 9.7 ± 7.8 days. It is worth underscoring that, for 10 of the 197 patients included in this study, it was not possible to calculate time from onset of symptoms to death ( Table 1 ).

The profile shown in this study is in agreement with that observed in other parts of the world.^3 - 5^ Nonetheless, the following three aspects warrant attention: i. the elevated proportion of individuals with multiple comorbidities (78.7% had two or more diseases/baseline risk factors), ii. the wide variety of diseases/risk factors observed, and iii. the clinical status in which individuals arrived at the hospital (compromised respiratory function).

The sum of comorbidities/risk factors in a single person may elevate the risk of mortality from COVID-19, although there are still not any precise estimates of these risks. In a study involving 416 hospitalized patients with COVID-19, carried out in a hospital in Wuhan, China, 44 (10.6%) and 22 (5.3%) had coronary heart disease and cerebrovascular disease, respectively. The also observed other comorbidities, such as chronic heart failure (4.1%; n = 17), chronic renal failure (3.4%; n = 14), chronic obstructive pulmonary disease (2.9%; n = 12), and cancer (2.2%; n = 9), which were also observed in our study. Mortality was higher in individuals with heart injury (51.2% in the group with injury vs. 4.5% in the group without injury), and preexisting diseases were factors associated with higher mortality.^4^

The association between several comorbidities/risk factors may explain the compromised respiratory condition upon admission, with dyspnea and oxygen saturation < 95%, which indicates severely compromised pulmonary function in these patients. The close functional relationship between the cardiovascular (doubly affected by the baseline disease and by SARS-CoV-2 infection) and pulmonary systems (accentuated pulmonary injury) should be prioritized during the process of caring for patients with COVID-19 who have circulatory system disease.^3 - 5^

Finally, we underline the need to adopt and/or strengthen mechanisms that reduce contamination of individuals with circulatory system diseases by COVID-19. For those who are already contaminated, early diagnosis and monitoring of clinical picture should be rigorously followed, in order to avoid worsening and death in these individuals.
